# Forelimb reduction and digit loss were evolutionarily decoupled in oviraptorosaurian theropod dinosaurs

**DOI:** 10.1098/rsos.242114

**Published:** 2025-03-26

**Authors:** Amelia Mead, Gregory Funston, Stephen Brusatte

**Affiliations:** ^1^ Department of Geology and of Geophysics, School of GeoSciences, University of Edinburgh, Edinburgh, UK; ^2^ Royal Ontario Museum, Toronto, Ontario, Canada; ^3^ University of California Davis, Davis, CA, USA

**Keywords:** dinosaur, Oviraptorosauria, digit loss, forelimbs, theropod, modularity

## Abstract

Theropod forelimbs exhibit wide morphological disparity, from the elongated wings of birds to the diminutive arms of *T. rex*. A wealth of work has sought to understand the evolution of bird flight via arm elongation, but despite widespread occurrences of forelimb reduction and digit loss throughout theropod dinosaurs, the evolutionary drivers behind these patterns are poorly understood. Previous studies demonstrate broad allometric trends that can account for some instances of forelimb reduction, but the repeated loss of digits, and their hypothesized link to forelimb shortening, has received less attention. Here, we evaluate evolutionary associations between digit loss and forelimb reduction in an iconic and data-rich theropod clade, Oviraptorosauria. Unexpectedly, we find that the evolution of digit III and the rest of the forelimb are decoupled. Support for different evolutionary models and a lower phylogenetic signal in digit III than the rest of the forelimb suggests these segments were subject to different evolutionary processes leading to independent morphological change. Oviraptorosaurs exhibit four distinct forelimb morphotypes, but these do not exactly correspond to patterns of dietary niche partitioning. Overall, forelimb evolution in oviraptorosaurs is more complex than anticipated, potentially as a result of an evolutionary radiation they underwent in the Late Cretaceous.

## 1. Introduction

Forelimb morphology is highly variable in tetrapods, but in almost all extant taxa, forelimb evolution is constrained by its role in quadrupedal locomotion [1–4]. Theropod dinosaurs are one of just a few tetrapod groups to evolve bipedality [1–4], with one group of derived theropods—Avialae (birds)—eventually going on to evolve powered flight [3,4]. For this reason, theropod limb evolution has attracted a great deal of study, the majority of which has focused on the evolution of long arms and flight in paravian theropods, a group which includes modern birds [5–8]. However, non-paravian theropod forelimbs also displayed dramatic morphological and functional variation [9–15], including repeated instances of forelimb reduction and digit loss [2,12,16], which have received less study.

In theropods, forelimb shortening is frequently attributed to allometry [7,17,18]. This is most famously discussed in Gould and Lewontin’s iconic ‘spandrels’ paper (1979), where the tiny forelimbs of *T. rex* were proposed to represent developmental ‘by-products’ of increased head and hindlimb (e.g. body) size. This kind of negative forelimb allometry has since been found to be pervasive across non-avian theropods [5–7] and has been suggested to play a crucial role in driving patterns of macroevolution within this group [7].

Digit loss also occurred repeatedly across non-avian theropods [9,10,16,18,19], and there are multiple instances where forelimb reduction is accompanied by a notable reduction or total loss of one or more digits. For example, alvarezsaurids [10,20], abelisaurids [2] and—most famously—tyrannosaurids [18,21,22] each show substantially shortened forelimbs with reduced or fewer digits. Thus, a macroevolutionary association between forelimb reduction and digit loss in theropods has been suggested, particularly where forelimb reduction is inferred to be linked to reduced functionality [7,19,23]. However, few theropod lineages have sufficient fossil records to test the link between forelimb reduction and digit loss, as intermediate morphologies are rare, and many clades underwent these transitions during the poorly sampled early Late Cretaceous [16,24,25]. Fortunately, it was recently recognized that a well-sampled clade underwent limb reduction and digit loss in the better-sampled latest Cretaceous: oviraptorosaurs [14].

Oviraptorosaurs were among the most diverse non-avian theropod clades, are known from abundant, relatively complete skeletons [13,14,23,26,27] and are hypothesized to have undergone an evolutionary radiation during the Late Cretaceous of Asia [14,26]. They possess a suite of bird-like characteristics not seen in more basal theropods, including parrot-like skulls with toothless beaks [13,26], crests with air-filled cavities [28,29], evidence of brooding behaviours [27,30–33] and some of the first recorded pennaceous feathers with a central quill and vanes [34,35]. Critically, a lineage of oviraptorosaurs, Heyuanninae, was recently recognized to have undergone forelimb reduction and digit loss, culminating in the functionally didactyl *Oksoko avarsan* [14,29]. Oviraptorosaurs, therefore, offer an ideal case study to evaluate the evolutionary links between forelimb reduction and digit loss.

Here, we use the excellent fossil record of oviraptorosaur theropods to evaluate key questions about the patterns of forelimb evolution in theropods: (i) Are forelimb reduction and digit loss evolutionarily integrated? (ii) What mode(s) of evolution best-fit forelimb evolution in a model theropod group? (iii) Could the evolution of the forelimb have contributed to the Late Cretaceous oviraptorosaur radiation?

## 2. Methods

### 2.1. Phylogenetic regression models

We augmented the dataset from Funston *et al*. [14] with measurements of other oviraptorosaurs recorded in the literature [23,36–38]. The resulting dataset included length measurements for the humerus, radius, ulna, metacarpals, manual phalanges, unguals, ungual curvature and femur for a broad range of oviraptorosaurs (51 different individuals across 25 different species). Both the linear and curved lengths were included for the unguals, as ungual curvature has been discussed as a functionally and ecologically important trait [39,40]. We then used the time-scaled phylogeny reported in Funston *et al*. [14] to fit a series of phylogenetic regressions testing for evolutionary associations between log-transformed values of humerus length and femur length (a proxy for body size), and between log-transformed humerus length and various manual elements (see electronic supplementary material). Phylogenetic regressions fitted to the relationship between humerus length and the length of each of the manual phalanges directly test whether the reduction of the forelimb is linked to a reduction in elements of digit III. Because there is evidence that forelimb reduction may affect the antebrachium to a greater degree than the humerus [41], to support the use of humerus length as a proxy for overall forelimb length, we also conducted a series of phylogenetic regressions testing for associations between log-transformed humerus plus radius length and elements of the manus. Following the results from PCA (see below), we also fitted a phylogenetic regression to two constructed variables: one consisting of the summed lengths of the humerus, radius, and metacarpal II and one consisting of the summed lengths of manual phalanges III−2 to 4.

We tested for significant differences between the slopes of each phylogenetic regression fitted to the relationship between the humerus and each of the proximal manual phalanges using Welch’s *t*-tests. These tests were also used to test for departures from isometry (slope of 1) in each of the proximal manual phalanges. All phylogenetic regressions were conducted using the package ‘phylolm’ [42]. 95% confidence intervals were computed for the slopes of each PGLS based on 1000 bootstrap replicates.

### 2.2. Principal component analysis

We culled the dataset to exclude any specimens missing values for more than 50% of measurements, resulting in a data frame of 25 individuals across 14 different species for our principal component analysis (PCA). As PCA cannot handle missing values, the package ‘missMDA’ [43] was used in R v4.2.2 [44] to impute values for missing data based on relationships between individuals. In brief, this package imputes missing values based on similar entries with more complete data, indirectly accounting for phylogenetic relatedness (for further details, see electronic supplementary material). Using this method, the imputed data should carry no weight and therefore not affect the results of downstream analyses [43,45]. To evaluate whether this method was appropriate for our dataset, multiple imputations were performed and compared to assess the uncertainty of the imputed estimates ahead of further analyses [43]. Initially, analysis of the multiple imputations indicated that PC2 tended to invert. To stabilize this axis and account for wide variation in body size, all observations were logarithmically transformed prior to imputation (electronic supplementary material, figure S5). To deal with measurements of 0 mm (e.g. for the distal manual phalanges of *Oksoko*), a value of 1.0 mm was added to every value in the dataset before the logarithmic transformation.

We used ‘FactoMineR’ [46] and ‘factoextra’ [47] to perform PCA. To assess whether the recovered PC axes were significant and which variables contributed the greatest loadings to each axis, we used the package ‘PCAtest’ [48]. The function ‘dimdesc()’ in the package FactoMineR [46] was used to assess which variables were significantly correlated with each PC axis. The PC1 and 2 coordinate values for one individual of each species were used to produce a phylomorphospace plot with ‘phytools v 1.5.1’ [49] and the phylogeny from Funston *et al*. [14].

### 2.3. Clustering analysis

The ‘elbow’ method with *k*-means clustering was used to define morphotype groups within Oviraptorosauria. This is an unsupervised machine-learning algorithm which can be used to group similar data points into clusters [50]. We ran a total of 10 k-means models and determined the within-cluster sum of squares (WCSS) for each model using between 1 and 10 clusters (*k* = 1–10). The total WCSS decreases as the value of *k* (the number of clusters) increases. We selected the model with the value of k for which the reduction of WCSS reached an elbow of inertia against the *k* curve—i.e. when the reduction in WCSS diminishes with increasing values of *k*. This was taken to be the optimum number of forelimb morphotype groups within Oviraptorosauria.

### 2.4. Assessing evolutionary mode

We used ‘phytools v 1.5.1’ [49] and the phylogeny from Funston *et al*. [14] to perform ancestral state reconstructions for selected continuous traits. These included PC1 and 2 coordinates, and the residuals from PGLS models fitted above. We then assessed the phylogenetic signal for PC1 and PC2 using Blomberg’s K [51]. Finally, four models of trait evolution—Brownian motion (BM), Ornstein–Uhlenbeck (OU), early burst (EB) and mean trend (MT)—were fitted to each of these traits and assessed using weighted AICc scores in the package ‘geiger’ [52].

## 3. Results

### 3.1. Phylogenetic regression models

**Figure 1.**
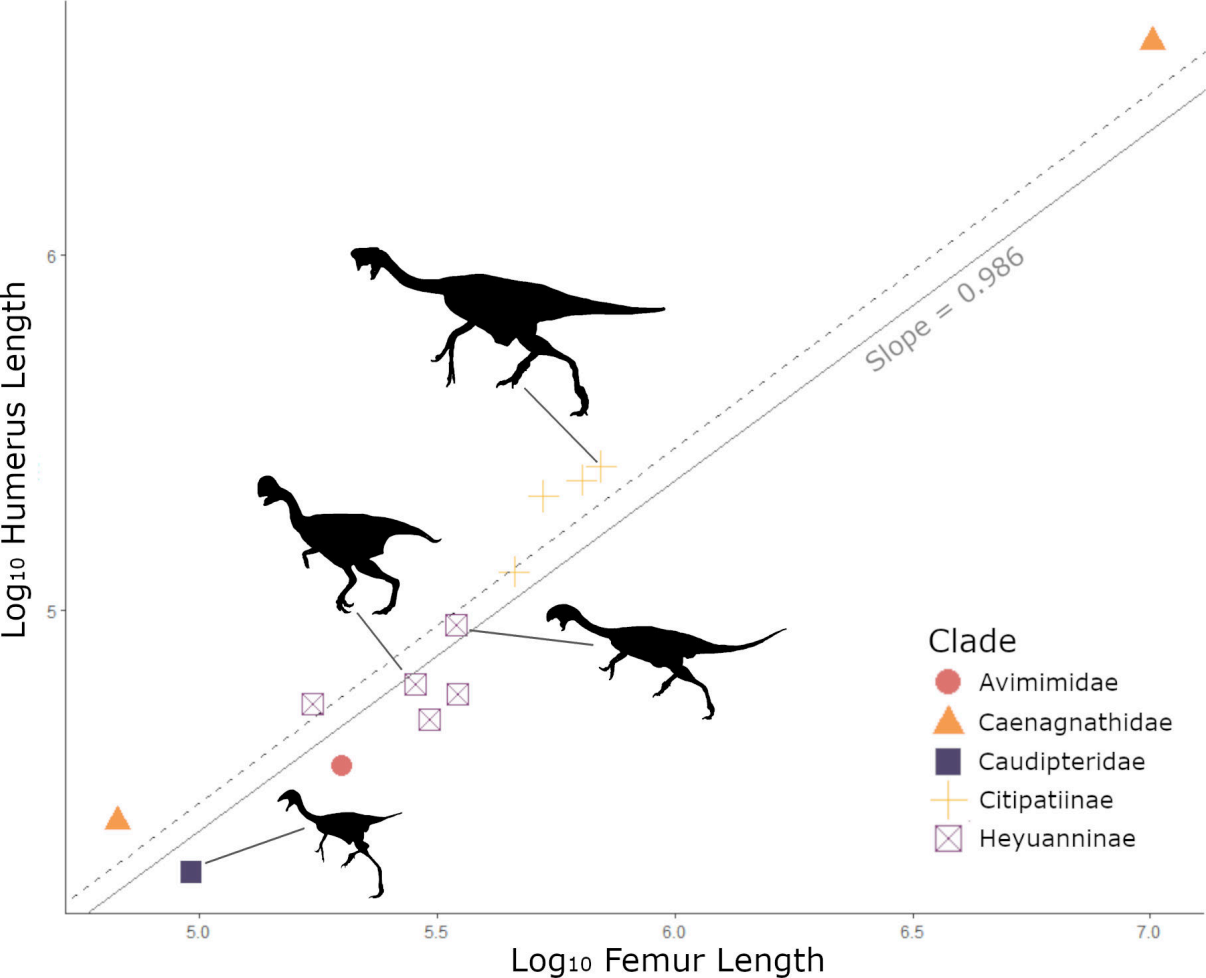
Relationship between humerus and femur length. Solid grey line shows PGLS regression model fitted, with slope indicated in grey. Dashed grey line represents slope expected under isometry, if humerus length were evolving in direct proportion to the femur length. Colours indicate different clades of oviraptorosaurs: avimimids (red), caenagnathids (orange), caudipterids (navy), citipatiines (yellow) and heyuannines (purple). Sketches of different oviraptorosaurs are in black, from bottom to top these represent *Caudipteryx zoui*, *Heyuannina yanshini*, *Oksoko avarsan* and *Citipati osmolskae*.

Humerus length was closely and significantly correlated with femur length (*R*^2^ = 0.947, *t* = 14.0156, 95% CI [0.966, 1.250], *p* = 0.000) (figure 1), and shows an approximately isometric relationship, as recovered in a previous analysis [53]. This differs from the positive allometric trend recovered in [14]. Different families of oviraptorosaurs appear to deviate slightly from the regression model applied here, although sample sizes are too small to do a series of individual subgroup regressions. For example, citipatiines and caenagnathids fall consistently above the regression line, regardless of femur length (figure 1), whereas heyuannines are more spread out, with some members of the group falling on either side of the regression line (figure 1). The sample size for both caudipterids and avimimids is too small to comment either way, with just a single taxon per group.

Both the humerus length and the combined lengths of the humerus and the antebrachium are significantly correlated with the lengths of all manual phalanges except the distal phalanges of digit III (table 1; electronic supplementary material, table S1 and figures S1 and S2). Although each regression model fitted for the relationship of the three proximal manual phalanges to humerus length has a different allometric coefficient (electronic supplementary material, tables S1 and S2), these all are statistically indistinguishable from isometry (electronic supplementary material, table S3) and from each other (electronic supplementary material, table S4).

**Table 1.** Correlations between log-transformed humerus length and manual phalanges. Asterisk (*) indicates significant correlations.

manual phalanx	*R* ^2^	*t*	*p*
I−1	0.8303	7.66358	0.00001*
I−2	0.8624	7.91815	0.00001*
II−1	0.7706	6.07872	0.00008*
II−2	0.4659	2.95359	0.01445*
II−3	0.6968	4.54743	0.00139*
III−1	0.6872	4.19247	0.00303*
III−2	0.1525	1.19965	0.2646
III−3	0.1957	1.39537	0.2004
III−4	0.1339	1.11234	0.2983

Finally, no significant correlation was found for the relationship between the added lengths of the humerus, radius, and metacarpal II and the added lengths of manual phalanges III−2 to 4 (*R*^2^ = 0.1711, *t* = 1.202, *p* = 0.268). For a full list of the slopes and intercepts of every regression model fitted, see supplementary material (electronic supplementary material, table S1).

### 3.2. Principal component and clustering analysis

**Figure 2.**
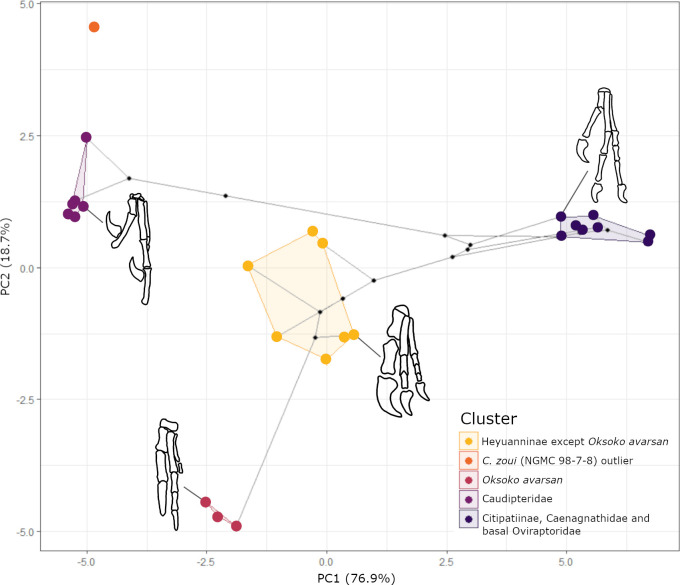
PCA plot, with phylogenetic relationships imposed, demonstrating morphological change in the oviraptorosaurian forelimb. Polygons correspond to clusters retrieved from *k*-means clustering analysis. Coloured dots represent oviraptorosaurs within the dataset, the larger coloured dots indicate individuals used to generate phylomorphospace. Small black dots represent ancestral state reconstructions at each node.

The first two PC axes only are significant, and between them, they explain 95.7% of the variation in the data (figure 2; electronic supplementary material, table S7). All 24 measurements of bone length have significant loadings on PC1, which accounts for 76.9% of the total variation (*p* = 0.000, 95% CI [71.9, 84.0] based on 1000 bootstrap replicates). The variables most closely correlated with this axis are the length of the ulna (*R*^2^ = 0.991, *p* = 0.00), the radius (*R*^2^ = 0.989, *p* = 0.00) and the metacarpal II (*R*^2^ = 0.989, *p* = 0.00). There are multiple other variables closely correlated with this axis, including the humerus (*R*^2^ = 0.977, *p* = 0.00). For a full list, see supplementary material (electronic supplementary material, table S5). Conversely, PC2 accounts for 18.7% of the total variation (*p* = 0.000, 95% CI [12.5, 23.0]) and has significant correlations with five variables: manual phalanx III−2 (*R*^2^ = 0.939, *p* = 0.00), III−3 (*R*^2^ = 0.912, *p* = 0.00), III−4 (*R*^2^ = 0.878, *p* = 0.00), the curved length of manual phalanx III−4 (*R*^2^ = 0.873, *p* = 0.00) and manual phalanx II−2 (*R*^2^ = 0.468, *p* = 0.00) (electronic supplementary material, table S6). Of these, only the distal phalanges of digit III had significant loadings on this PC (electronic supplementary material, table S6). Therefore, the two significant PC axes represent the whole forelimb length and the length of the distal phalanges of digit III, respectively—high values for PC1 indicate longer forelimbs, and high values for PC2 indicate a longer third digit. We tested the sensitivity of the PCA to data imputation and found minimal error with no consistent skew, suggesting these results are not strongly influenced by the imputed estimates (electronic supplementary material, figures S3–S6).

*k*-Means clustering analysis grouped oviraptorosaurs into five groups across this morphospace (figure 2; electronic supplementary material, figure S7), however from this, we interpret only four distinct forelimb morphotypes. This is because one group contains just a single specimen of *Caudipteryx zoui* (NGMC 98−7−8), which seems to be clustering independently from all other *C. zoui* specimens in the dataset. All other members of Caudipteridae cluster relatively tightly together in the top left of the plot (figure 2), with low values for PC1 and high values for PC2; members of this group are therefore characterized by relatively short forelimbs and potentially long third digits. There is a high degree of uncertainty around the imputations for digit III measurements in these taxa (electronic supplementary material, figures S3 and S4) resulting from the large proportion of missing data for the length of manual phalanges III−1 to III−4 in caudipterids (more detail in electronic supplementary material). However, the error ellipse strongly overlaps with those of other members of Caudipteridae, indicating it is not significantly different from them. *k*-Means clustering is limited in that it cannot take into account the degree of uncertainty around the position of NGMC 98−7−8 resulting from data imputation, causing it to recover this specimen as its own group, distinct from other specimens of the same species. Given the substantial degree of overlap in the errors around the position of all members of Caudipteridae, we interpret this clade as a single morphotype group.

There are three other distinct forelimb morphotype groups, which are recovered as separate clusters by *k*-means analysis. Early-branching oviraptorids, including *Yulong mini* [23] and *Oviraptor philoceratops* [54], members of the subfamily Citipatiinae, and the single caenagnathid taxon, *Apatoraptor pennatus* [55], cluster as a paraphyletic group in the top right, with high values for PC1 and slightly lower values for PC2 relative to the caudipterids (figure 2). This corresponds to a long digit III and longer forelimbs than other oviraptorosaurs. Members of the subfamily Heyuanninae are split into two distinct groups. The majority fall into a relatively broad, central group (figure 2), with intermediate values on both PC1 and PC2. This corresponds to mid-length forelimbs compared to other oviraptorosaurs with variation in the degree of digit III reduction within members of this group. The second heyuannine group consists only of *Oksoko* specimens, which have slightly reduced values for PC1 relative to other heyuannines and extremely reduced values for PC2 (figure 2). This is indicative of slightly shorter forelimbs—although not as highly reduced as in the Caudipteridae cluster—and the highly reduced third digit.

### 3.3. Assessing evolutionary mode

**Figure 3.**
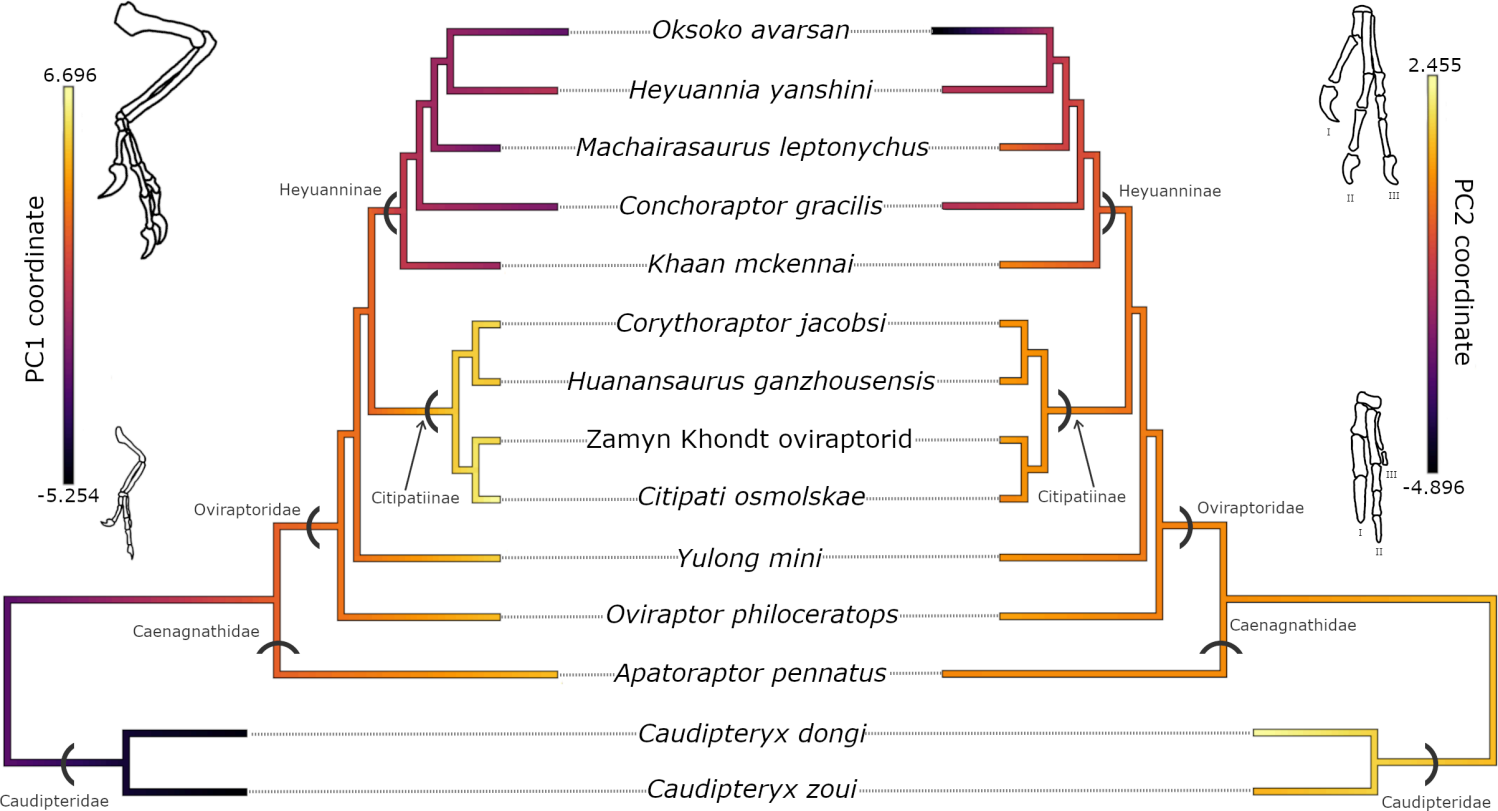
Ancestral state reconstruction of traits associated with PC1 (left) and PC2 (right) throughout the oviraptorosaurian phylogeny.

**Figure 4.**
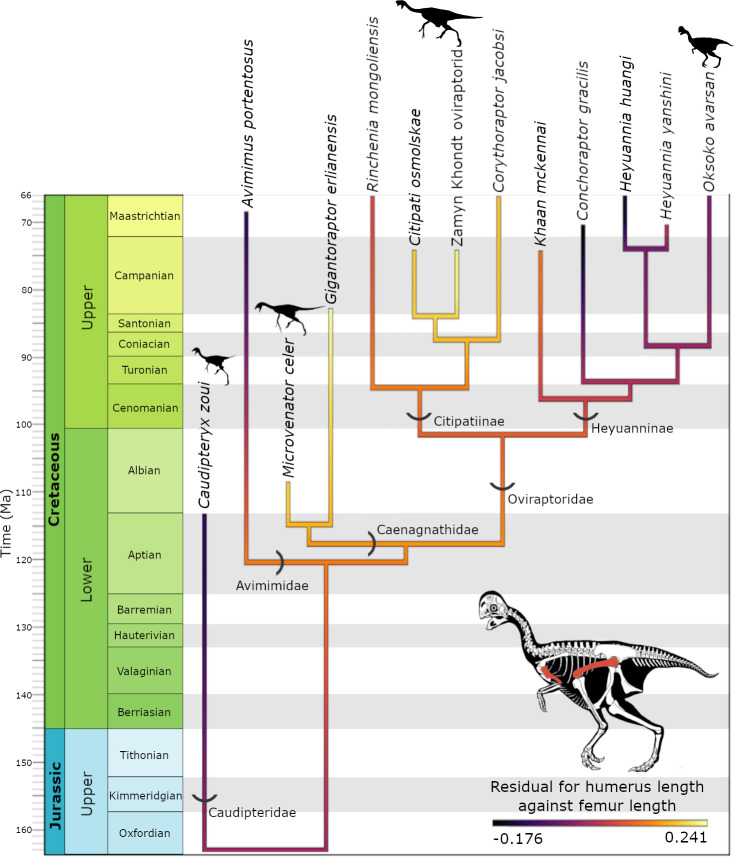
Ancestral state reconstruction of the residuals of the relationship between humerus and femur length from PGLS through the oviraptorosaurian phylogeny.

Ancestral state reconstructions for PC1, representing overall forelimb length, show low initial values for caudipterids at the base of Oviraptorosauria, before increasing in more derived oviraptorosaurs, like caenagnathids and citipatiines (figure 3). This is followed by a reduction in PC1 at the base of Heyuanninae. All heyuannines analysed show similar, mid-range values for PC1. In the heatmap for the residuals of the relationship between humerus and femur length (figure 4), the pattern appears to be broadly similar to PC1, with relatively short humeri in early oviraptorosaurs like *C. zoui* and longer humeri in caenagnathids and most citipatiines. However, whilst derived citipatiines appear to have elongated humeri, the more basal citipatiine, *Rinchenia mongoliensis*, exhibits humerus reduction similar to *Oksoko*. Broadly, Heyuanninae records a strong reduction in humerus length, similar to PC1, with two heyuannine taxa—*Conchoraptor* and *Heyuannia huangi*—recording exceptionally low values for the humerus : femur length residuals. These taxa had much shorter humeri than expected based on the length of their femur. Intriguingly, it is these taxa—and not *Oksoko*—that show the greatest reduction in humerus length relative to femur length.

The evolution of PC2, correlated with the distal manual phalanges of digit III, follows a different pattern than PC1 (figure 3). Caudipteridae show relatively high values for PC2, which steadily decrease into Caenagnathidae and Citipatiinae. There are several slight, seemingly independent, reductions of PC2 values in Heyuanninae, in *Conchoraptor*, *Heyuannia yanshini*, and a dramatic decrease in *Oksoko avarsan* (figure 3).

The lengths of the manual phalanges of digit III, corrected for humerus length, mostly follow a similar trend to PC2, whereby Caenagnathidae and Citipatiinae demonstrate high trait values followed by a decrease throughout Heyuanninae that culminates in extreme reduction in *Oksoko* (electronic supplementary material, figure S8). There is one key exception to this trend; *Heyuannia yanshini* exhibits a degree of reduction in manual phalanx III−1 relative to the humerus similar to that of *Oksoko*. However, neither manual phalanx III−2 nor III−3 are similarly reduced in *H. yanshini*. Another interesting exception is *Apatoraptor pennatus*, a caenagnathid which shows moderate reduction of manual phalanx III−3, similar to the heyuannines *Khaan mckennai* and *Conchoraptor gracilis*.

Phylogenetic signal was significantly stronger than expected under Brownian motion (BM) for both PC1 and significantly weaker than expected for PC2 (table 2). The only model which receives strong support in this analysis is the BM model for the evolution of PC1 (table 3). Although mean-trend (MT) is the best-supported model of evolution for PC2, this support is weak and not sufficient to conclusively support this as the best explanation for the evolution of this axis and its associated traits.

**Table 2.** Measures of phylogenetic signal in different traits. Significant values are marked with an asterisk (*).

trait	transformation	Blomberg’s *k*	***p*-value**
PC1	none	1.93337*	0.001
PC2	none	0.829527*	0.033

**Table 3.** Weighted AICc scores for each of the four evolutionary models tested for each different trait. These were BM representing a random walk, Ornstein–Uhlenbeck (OU) representing a random walk with evolutionary bounds, an Early Burst (EB) model representing a decelerating rate of evolution through time, and the mean-trend (MT) model representing a random walk with directional change [42]. Models with notably greater support are marked with (*).

trait	weighted AICc score for each model			
	BM	OU	EB	MT
PC1	0.5313567*	0.1016274	0.1276545	0.2393615
PC2	0.34068160	0.06513116	0.06512554	0.52906171

## 4. Discussion

### 4.1. Forelimb reduction and digit loss are not tightly evolutionarily correlated

Contrary to expectations [23], forelimb reduction and the loss of digit III are not closely evolutionarily correlated in oviraptorosaurs (figure 2). Instead, we find strong evidence for decoupled evolution between digit III and the rest of the forelimb [48,56,57]. The length of the ulna, the radius, metacarpal II and the humerus contribute most strongly to PC1, indicating tight evolutionary associations between these bones but weaker associations with those which contribute to PC2—manual phalanges III−2 to 4. Phylogenetic regressions (PGLS) further support decoupled evolution between digit III and the rest of the forelimb. Humerus length was not significantly correlated with the lengths of any of the distal elements of digit III (manual phalanges III−2 to 4) (electronic supplementary material, figure S1b) but was significantly correlated with the lengths of all other manual phalanges (table 1). The inability of humerus length to predict the length of manual phalanges III−2 to III−4 further indicates there is no significant evolutionary association between these traits [58]. The decoupled evolution between the distal elements of digit III and the rest of the forelimb can be explained by the presence of two evolutionary modules [56,57,59–62]. One module consists of the distal elements of digit III, associated with PC2 (figure 3), and the other consists of the more proximal forelimb elements associated with PC1 (figure 3).

Previous studies have shown that tetrapods generally have two evolutionary modules in their forelimbs; but this division is usually drawn between all of the digits and the remainder of the forelimb [41,61,63–65]. Our results therefore exhibit some key differences from broader tetrapod trends. First, all the manual phalanges of digits I and II, and the proximal manual phalanx of digit III, are significantly correlated with humerus length (table 1; electronic supplementary material, figures S1a and S2), supporting an evolutionary association between these elements [58] and suggesting these all form part of the proximal forelimb module in oviraptorosaurs. Second, only the distal elements of digit III appear to be decoupled from the rest of the forelimb, rather than the entire distal autopod [41,63]. Critically, we find that the evolution of digit III was decoupled from other digits, suggesting that not all digits were part of the same evolutionary module in oviraptorosaurs. This discrepancy could be because patterns of modularity and integration are themselves subject to selection pressures and therefore can evolve like any other trait [61]. This might be particularly relevant with respect to bipedality in theropods, in the sense that selection on forelimb modularity might differ from other tetrapods where the forelimb is typically used in locomotion. Alternatively, this could reflect the semi-autonomous nature of evolutionary modules—some interdependence frequently exists across segments which would otherwise evolve separately [57].

Also reflective of the semi-autonomous nature of evolutionary modules is the weak but significant correlation of the distal elements of digit III with PC1 (electronic supplementary material, table S5). These relationships are characterized by low *R*^2^ values (electronic supplementary material, table S5), indicating that, although these correlations are significant, they are only able to account for a small portion of the variability observed in the distal phalanges of digit III. Conversely, the correlation between PC2 and each of the distal digit III phalanges is both significant and able to explain the majority of variability in these traits (electronic supplementary material, table S6). Coupled with the absence of significant correlations between forelimb length and the length of any of the distal phalanges of digit III (table 1; electronic supplementary material, table S2), this is strong evidence that the distal part of digit III can be considered its own evolutionary module. However, modularity and integration are not binary and occur on a spectrum [41,57,63]. Organisms are generally accepted not to be strictly modular: although traits within a single module are highly integrated with each other and relatively little integration exists between traits in separate modules, some degree of integration does usually still exist between the otherwise ‘independent’ modules [56,57,59–62,66]. The weak correlation of the distal elements of digit III with PC1 agrees with this interpretation, and indicates some degree of integration does exist between the evolutionary modules identified here.

Although there are many examples of the concurrent loss of digit III and the reduction of the forelimb in tetanuran theropods [19,22,25,67], other instances of digit loss occurring earlier in theropod phylogeny are not associated with highly reduced forelimbs or a potential loss of function [12,68–71]. For example, *Herrerasaurus ischigualastensis* had comparatively long forelimbs, likely adapted for prey capture and manipulation [69], despite the reduction of digits IV and V to vestigial structures [12,68,69]. Furthermore, a vestigial fourth digit has been observed in early members of Tyrannosauroidea, and Maniraptora [68], without an accompanying dramatic reduction of forelimb length: the early tyrannosauroid, *Guanlong wucaii*, and the early maniraptoran, *Ornitholestes hermanni*, both show extreme reductions of digit IV whereby metacarpal IV is reduced to a spherical and ‘featureless’ vestige [68,70]. In both these taxa, the forelimb is relatively long [70,71] and, in *Ornitholestes*, it has been associated with a grasping function [71]. Combined with the decoupled evolution of digit III from the rest of the forelimb, recovered here in oviraptorosaurs, the fossil record indicates that digit loss in theropods is not always linked to a concurrent reduction of the forelimb as a whole or even a loss of function. This might indicate an ancestral modularity pattern within theropods, whereby the whole distal autopod is decoupled from the rest of the forelimb, as in the rest of Tetrapoda [41,63].

### 4.2. Two distinct patterns of forelimb evolution

Our results provide strong evidence that not all parts of the forelimb were evolving under BM: the distal elements of digit III do not follow the same pattern of evolution as the overall length of the forelimb (figure 3, table 3). The reduction of digit III in heyuannine oviraptorids, reflected in the PCA (figure 2) and ancestral state reconstructions (figure 3), is accompanied by a departure from the expectations of BM (tables 2 and 3). This departure is indicated by a low phylogenetic signal [51] and a lack of support for the BM model of evolution in PC2, which is most tightly correlated with the manual phalanges III−2 to 4. Instead, we find relatively weak support for an evolutionary model of mean trait change over time (table 3). This contrasts with PC1, representing the rest of the forelimb, for which we found a significantly high phylogenetic signal (table 2) and strong support for the BM model of evolution (table 3).

The strong support for BM in the evolution of PC1 (table 3) indicates that whole-forelimb length evolved under random drift [72]. However, given that the phylogenetic signal for PC1 is significantly greater than 1 (table 2), in this instance, support for the BM model might be an oversimplification of more complex evolutionary patterns. There are multiple evolutionary scenarios which can elevate phylogenetic signal, including evolution under BM but with a variable rate [51,73]. This typically occurs when evolutionary rate begins high and decreases towards the present, for example, as ecological niches saturate during an adaptive radiation [51,73]. This explanation for the high phylogenetic signal is consistent with the exceptionally taxonomically rich fossil record of oviraptorosaurs [13,14,23,26,27], which has been suggested to reflect an evolutionary radiation during the Late Cretaceous of Asia [26]. A recent study has found evidence for high evolutionary rates in non-paravian dinosaurs, including oviraptorosaurs [8], potentially supporting this idea. However, future studies investigating more complex evolutionary models for the proximal forelimb module in oviraptorosaurs, including niche-filling models consistent with adaptive radiations [73], are needed to more directly test this hypothesis.

In contrast, the MT model was preferred for PC2 (table 3). However, AICc and AICc-weighted scores indicate this support is relatively weak. The MT model is similar to BM, except that the mean of each evolutionary ‘step’ is non-zero [72], resulting in directional evolution. Although support for this model is not conclusive, the significantly low phylogenetic signal recovered for PC2 supports a departure from the expectations of BM. There are several potential explanations for low phylogenetic signals, for example, when trait evolution (i) is under strong stabilizing selection; (ii) is bounded with a high or fluctuating evolutionary rate; or (iii) is under divergent selection [51]. Of these, our results are most consistent with divergent selection. In particular, phylogenetic signals in traits evolving under strong stabilizing selection are usually much lower than that recovered for the distal elements of digit III, with values close to zero [51]. If the low phylogenetic signal for PC2 were caused by bounded evolution, for example by constant functional constraint, under high or fluctuating evolutionary rates, we would expect to find support for the Ornstein-Uhlenbeck (OU) model of evolution [51,72]. However, we do not find any support for the OU model in PC2 (table 3). It is therefore unlikely that the distal elements of digit III are evolving under strong stabilizing selection or some other form of bounded evolution. The preference for the MT model for PC2 might therefore indicate the low phylogenetic signal is driven by directional evolution linked to divergent selection. However, support for this model is not conclusive and therefore caution should be applied to this interpretation.

Previous studies have suggested that digit III reduction in Heyuanninae could have evolved in response to novel selection pressures encountered as they expanded into the Gobi Desert from Southern China [14]. Visible patterns in the PC and phylomorphospace plot (figure 2) and in ancestral state reconstructions (figure 3) indicate the reduction of digit III began at the base of Heyuanninae, independently of the reduction of the forelimb as a whole. The reduction of the digit was gradual and increased through the evolution of heyuannines, such that, based on the phylogeny of [14], successively branching heyuannines had progressively shorter third digits (electronic supplementary material, figure S9). Contributing to this pattern, phalanx III−3, which is typically longer than III−2 in oviraptorosaurs, appears to decrease in relative length throughout the heyuannine radiation: phalanx III−3 is longer than III−2 in *Khaan*, subequal in *Machairasaurus*, and shorter than III−2 in *Heyuannia yanshini* [13,74,75]. Considering that longer penultimate phalanges have a beneficial effect on grasping functions [64,76,77], the reversal in this pattern in heyuannines may suggest that selective pressures for grasping were relaxed in digit III and that this digit became subject to selection for other functions (or none at all). Combined with the significantly low phylogenetic signal for PC2, our results do not contradict the hypothesis that digit III reduction could be an evolutionary response to exposure to different selection pressures following range expansion into the Gobi Desert [14]. We agree it is possible that novel selection pressures could have caused the heyuannine digit III to evolve in a different direction than other oviraptorids and this interpretation would be consistent with the MT model of directional evolution. However, due to the relatively weak support for this model, future analyses—for example, into evolutionary rates within Oviraptorosauria—are necessary to provide additional evidence with which to evaluate this hypothesis.

### 4.3. Four distinct morphotype clusters

Oviraptorosaurs cluster into four distinct forelimb morphotypes (figure 2). Previously, morphological differences in the oviraptorosaur forelimb have been linked to shifting functions in food acquisition: the elongated manus and forelimb of caenagnathids has been associated with grasping prey [9,13], and the reduced forelimbs of some oviraptorids has been used as evidence for herbivory and a loss of predatory function [13]. However, there appears to be a mismatch between forelimb morphotype groups and cranial functional morphology [78]. For example, caenagnathids are significantly separated from oviraptorids in lower beak form and in mandible form and function [78], but fall within the same morphotype cluster as Citipatiinae, members of Oviraptoridae (figure 2). If dietary niche was the primary driver of forelimb evolution in oviraptorosaurs, we would expect oviraptorosaurs with functionally divergent skulls to also have functionally divergent forelimbs, but this does not appear to be the case (figure 2) [78]. Thus, although separation in cranial functional morphology between caenagnathids and citiapatiines suggests they occupied different dietary niches [13,78], their forelimbs may more closely reflect a shared a conserved function, for example their use in brooding behaviour [27,31,32,79,80].

Conversely, within oviraptorids, the crania are relatively similar in terms of their functional morphology [78] and biomechanics [28], but oviraptorid forelimbs exhibit broad variation, falling into three distinct morphotype clusters (figure 2). Citipatiines and other, earlier-branching, oviraptorids cluster with caenagnathids with an elongated forelimb and manus (figure 2), whereas members of Heyuanninae are divided across two separate clusters (figure 2). There is wide variation in digit III length across Heyuanninae, becoming increasingly reduced in derived heyuannines and culminating in the vestigial splint seen in *Oksoko* (figure 2) [14]. There is also a marked reduction in PC1 at the base of Heyuanninae (figure 2), indicating a reduction in the length of the humerus, radius, ulna and metacarpal II relative to citipatiines, caenagnathids and more basal oviraptorids. From what is currently known of the oviraptorosaur fossil record, this reduction in both digit III and the forelimb overall appears to coincide with the expansion of Heyuanninae into the Gobi Desert from Southern China [14]. Because this reduction seems to have occurred after the range expansion, it is possible selective pressures in this novel environment caused a shift in forelimb function which ultimately selected for the reduction of digit III and the forelimb [14].

Alongside *Oksoko* and multiple species of oviraptorosaurs [13,14], the Nemegt and Baruungoyot Formations, which are different lithologies of the same ecosystem [13,81], have yielded numerous species of alvarezsaurids [82–88]. Parvicursorine alvarezsaurids, including *Mononykus olecranus* and *Nemegtonykus citus* from the Nemegt Formation, have reduced limbs and have lost or significantly reduced multiple digits, with digit II becoming notably wider and deeper [20,82,83]. Combined with other features of the forelimb, including a distally located deltopectoral crest, a large olecranon process on the ulna, and a shortened forelimb and manus, the forelimb of these dinosaurs has been likened to those of extant mammalian taxa and interpreted as adapted for digging [10,20,89]. A recent description of the osteology of *Oksoko avarsan* noted an expanded deltopectoral crest, ectepicondylar tuber, and medial process of the ulna, indicative of strong musculature, as well as a more robust digit I than other oviraptorosaurs, topped with a trenchant ungual [29]. It has been suggested the forelimb could therefore have been similarly adapted to scratch digging—for example in foraging or nest building [29]. It is possible that use of the forelimb in scratch digging resulted in a selective advantage in oviraptorosaurs which had reduced third digits and more robust first digits. However, detailed myological reconstructions and range-of-motion analyses are necessary to test this hypothesis [14,29].

### 4.4. Forelimb evolution and the oviraptorosaur radiation

Oviraptorosaurs, particularly oviraptorids, were noticeably diverse throughout the Late Cretaceous compared to other theropods from this period [13,14,26,90]. It has been suggested that an evolutionary radiation [26], possibly associated with palaeogeographic factors such as rare dispersal events between geographically separated oviraptorid communities in southern China and the Gobi Desert [14], could have resulted in the remarkable diversity of oviraptorids seen in the fossil record [91–93]. The high phylogenetic signal we recover for PC1 (representing whole-forelimb length) could be interpreted as consistent with this hypothesis. Some recent work has found that non-paravian theropods, including oviraptorosaurs, have high limb disparity compared to paravians and elevated evolutionary rates in the forelimb compared to other theropods [8]. The pattern of forelimb modularity uncovered here may therefore have played a part in the evolution of increased forelimb disparity in oviraptorosaurs by allowing the exploration of novel forelimb morphospace. This could have been a contributing factor in the evolutionary radiation of oviraptorosaurs throughout the Late Cretaceous, especially given the association of digit III reduction with the range expansion of Heyuanninae into the Gobi Desert [14], which suggests this may be an adaptation of the forelimb for a new environment. Whether oviraptorosaurs were distinct in their forelimb adaptability could be tested by analysing other theropod groups to determine whether oviraptorosaurs show significantly different patterns of modularity, disparity and evolutionary rates.

In addition to their exceptional diversity, it is important to note that many oviraptorosaur species coexisted in Late Cretaceous ecosystems [13,94]. It has been suggested that variation in mandibular functional morphology [78], bite force [28] and body size [28,95] enabled niche partitioning between co-existing oviraptorosaurs. Alongside these, shifts in forelimb morphology and their associated function in heyuannine oviraptorids could have generated an additional dimension of niche partitioning in areas where multiple species of oviraptorosaurs co-existed, like southern China and the Gobi Desert [13,14]. For example, the reduced forelimbs of *Oksoko* could have been used for scratch-digging, perhaps in foraging or nest-building [29], allowing them to exploit different ecological resources than the caenagnathid, *Elmisaurus rarus*, found in the same formation, which had an elongated manus, better adapted for grasping [13,96]. The ability to take advantage of novel ‘ecological opportunities’, including resources or ecological niches, is necessary for evolutionary radiations to occur following the dispersal of a species into a new environment [91–93]. Modularity in the forelimb could have allowed species of oviraptorosaurs to take advantage of novel ecological opportunities and diversify following palaeogeographic expansion into the Gobi Desert. It is possible, therefore, that the patterns of modularity in oviraptorosaurian forelimbs contributed to the notable taxonomic and morphological diversity of these animals through the Late Cretaceous, either by enabling further niche partitioning, contributing to an evolutionary radiation, or both.

Our results highlight the remarkable complexity of forelimb evolution in Oviraptorosauria, including the surprising result that digit loss was not intrinsically linked to forelimb reduction in this clade. Instead, we find decoupled evolution between the distal digit III and the rest of the forelimb, resulting in four forelimb morphotypes (figure 2), which do not seem to be related to diet, based on discrepancies with cranial morphology [78]. Phylogenetic signal in the forelimb is consistent with an evolutionary radiation in oviraptorosaurs [26,51,73] and enhanced forelimb disparity appears to correspond to niche partitioning [13,14]. These observations can be explained by the hypothesis that evolutionary modularity in the forelimb, which facilitated an additional dimension of niche partitioning, coincided with an evolutionary radiation of oviraptorosaurs in the Late Cretaceous of Asia. We recommend further assessment of evolutionary rates, as well as biomechanical studies and myological reconstructions of the oviraptorosaur forelimb, to more fully test this hypothesis. Overall, our results suggest that the adaptability of the forelimb may have contributed to an evolutionary radiation of oviraptorosaurs in Asia, one of the last major dinosaur diversifications before the end-Cretaceous mass extinction.

## Data Availability

All data used in this study can be found in the supplementary material [97].
